# Blood volume in patients likely to be preload responsive: a post hoc analysis of a randomized controlled trial

**DOI:** 10.1186/s40635-023-00500-y

**Published:** 2023-03-31

**Authors:** Anja Lindén, Svajunas Statkevicius, Johan Bonnevier, Peter Bentzer

**Affiliations:** 1 Anesthesiology and Intensive Care, Department of Clinical Sciences Lund, Helsingborg Hospital, Lund University, Helsingborg, Sweden; 2 Intensive and Perioperative Care, Department of Clinical Sciences Lund, Skane University Hospital, Lund University, Lund, Sweden

**Keywords:** Blood volume, Postoperative patient, Preload, Preload responsiveness

## Abstract

**Background:**

Preload responsive postoperative patients with signs of inadequate organ perfusion are commonly assumed to be hypovolemic and therefore treated with fluids to increase preload. However, preload is influenced not only by blood volume, but also by venous vascular tone and the contribution of these factors to preload responsiveness in this setting is unknown. Based on this, the objective of this study was to investigate blood volume status in preload-responsive postoperative patients.

**Methods:**

Data from a clinical trial including postoperative patients after major abdominal surgery were analyzed. Patients with signs of inadequate organ perfusion and with data from a passive leg raising test (PLR) were included. An increase in pulse pressure by ≥ 9% was used to identify patients likely to be preload responsive. Blood volume was calculated from plasma volume measured using radiolabelled albumin and hematocrit. Patients with a blood volume of at least 10% above or below estimated normal volume were considered hyper- and hypovolemic, respectively.

**Results:**

A total of 63 patients were included in the study. Median (IQR) blood volume in the total was 57 (50–65) ml/kg, and change in pulse pressure after PLR was 14 (7–24)%. A total of 43 patients were preload responsive. Of these patients, 44% were hypovolemic, 28% euvolemic and 28% hypervolemic.

**Conclusions:**

A large fraction of postoperative patients with signs of hypoperfusion that are likely to be preload responsive, are hypervolemic. In these patients, treatments other than fluid administration may be a more rational approach to increase cardiac output.

*Trial registration* EudraCT 2013-004446-42

## Background

Signs of inadequate organ perfusion in the postoperative phase after major surgery are often treated with fluid to increase preload. Given that overly aggressive fluid treatment is likely to have adverse effects, guidelines suggest that prediction of fluid responsiveness by dynamic indices should be used to identify patients that are likely to respond to fluid therapy with an increase in cardiac output; in other words, patients on the steep part of the Frank–Starling curve [1].

The physiological rationale for treating all preload-responsive patients with signs of inadequate organ perfusion with fluids could, however, be questioned. The volume of the venous circulation can be divided into the unstressed and stressed volumes. The unstressed volume is the volume that fills the vessel without exerting pressure on the vessel walls. The stressed volume is the volume that distends the veins and creates the mean systemic filling pressure (*P*_msf_) which, together with right atrial pressure, are the major determinants of venous return and hence preload [2]. Because stressed venous volume is dependent on both venous tone and on blood volume it could be argued that the appropriate intervention to increase preload is dependent on the blood volume status of the patient [3]. We are not aware of any studies investigating the blood volume status in patients that are likely to be preload responders.

Based on the above, the objective of this study was to investigate blood volume status in preload-responsive postoperative patients. For this purpose, we analyzed blood volume in a cohort of postoperative patients after major abdominal surgery. Patients likely to be preload responders were identified by assessing change in arterial pulse pressure after a passive leg raising test.

## Methods

The study is a post hoc analysis of data collected in the albumin infusion rate and plasma volume expansion (AIR) trial. AIR was a single-center, investigator-initiated prospective parallel-group, randomized trial designed to assess if plasma volume expansion by 5% albumin is influenced by infusion rate after major abdominal surgery. The main results have been published elsewhere [4]. Briefly, adult patients scheduled for Whipple’s procedure due to pancreatic cancer or major gynaecological cancer surgery were approached prior to the operation and consent was obtained prior to the start of the operation. Included patients received routine pre- and intraoperative care. Anesthesia was induced with propofol and maintained using either sevoflurane or desflurane. Patients received an epidural catheter for intra- and post-operative analgesia unless contraindicated. All patients received an arterial line, calibrated according to manufacturer’s instruction, with a zero reference at the level of the anterior axillary line. Postoperatively, all patients were extubated and epidural analgesia was provided using bupivacaine (2.5 mg/ml) and morphine (0.05 mg/ml) at a rate of 4–6 ml/h. Crystalloids and colloids were used as resuscitation fluids intraoperatively at the discretion of the attending anesthetist. Postoperatively, patients with suspected hypovolemia were included in the AIR study and a plasma volume measurement was performed.

Ethical approval was provided by the regional ethical vetting board in Lund, Sweden (# 2014/15) and all patients gave written consent. The AIR study was registered in the European Clinical Trials Database (EudraCT 2013-004446-42).

### Inclusion criteria

Patients in the AIR database were included in the present study if:

1. One or more signs of hypoperfusion was observed within 5 h of admission to the post-anesthesia care unit. Signs of hypoperfusion were defined as:
  - ScvO_2_ < 70%,
  - lactate > 2 mmol/l,
  - urine output < 0.5 ml/kg in the hour prior to inclusion,
  - systolic BP < 100 mmHg or mean arterial pressure (MAP) < 55 mmHg.
2. Complete data from a passive leg raise test were available.
3. A plasma volume measurement performed within 30 min after the passive leg raise test was available.

All measurements were thereby performed within 5.5 h of admission to the post-anesthesia care unit.

### Passive leg raise test

The passive leg raising test reversibly increases preload by transferring blood from the lower extremities to the central compartment and meta-analyses suggest good discrimination between preload responders and non-responders in a large number of studies [5–7]. At the beginning of the test the patient was placed in a 45-degree head-up semi-recumbent position. Following recording of baseline pulse pressure, the upper body was lowered to horizontal position and legs were raised to 30 degrees. Pulse pressure was then assessed again. The highest value within 2 min of the PLR was recorded. A pulse pressure increase ≥ 9% was used to identify patients likely to be preload responsive [8].

### Blood volume

Blood volume (BV) was calculated from baseline plasma volume (PV) and hematocrit (Hct). The formula used was [9]:

$${\text{BV}} = {{{\text{PV}}} \mathord{\left/ {\vphantom {{{\text{PV}}} {\left( {{1} - {\text{Hct}}} \right)}}} \right. \kern-0pt} {\left( {{1} - {\text{Hct}}} \right)}}.$$

PV was measured using ^125^I human serum albumin (HSA) (SERALB-125^®^) as described in detail previously [4]. Briefly, a known dose of ^125^I-HSA was injected intravenously and the concentration of ^125^I-HSA in plasma at 10 min post-injection was measured using a gamma counter. Plasma volume was then calculated by dividing the injected dose of ^125^I-HSA by the change in concentration of ^125^I-HSA in plasma at 10 min post-injection and was indexed according to patient weight. Hematocrit was measured by colorimetric analysis using a blood gas analyser (Radiometer 850; Radiometer, Copenhagen, Denmark). Since large vessel hematocrit is higher than the body hematocrit, the measured Hct-value was corrected by multiplying with 0.9 [10]. The measured BV was then compared to the predicted normal BV, derived from height and weight of the patient [11, 12]. Precision of blood volume measurement using the present methodology is suggested to be ± 5% and patients were considered hypovolemic if the measured BV was at least 10% lower than the predicted normal BV, as previously suggested [13]. Patients were considered hypervolemic if measured BV was at least 10% higher than the predicted normal BV. Euvolemic patients were defined as patients with a BV deviating less than 10% from predicted normal BV. Hemodynamic data and hematocrit were recorded regularly for 3 h after the plasma volume measurement and at the end of this period, transcapillary escape rate of ^125^I-HSA was measured to assess vascular leak [4].

### Statistics

The analysis plan was determined prior to performing the analysis. No power calculation was carried out; number of available patients determined sample size. Continuous variables were analyzed using descriptive statistics, and categorical data using proportions. Correlation analysis was performed using Spearman’s correlation for non-parametric data. A *p*-value < 0.05 was considered to reflect statistical significance. All analyses were performed using PRISM 9.0.0 (Graphpad Software Inc., La Jolla, CA, USA).

## Results

### Study cohort

**Fig. 1:**
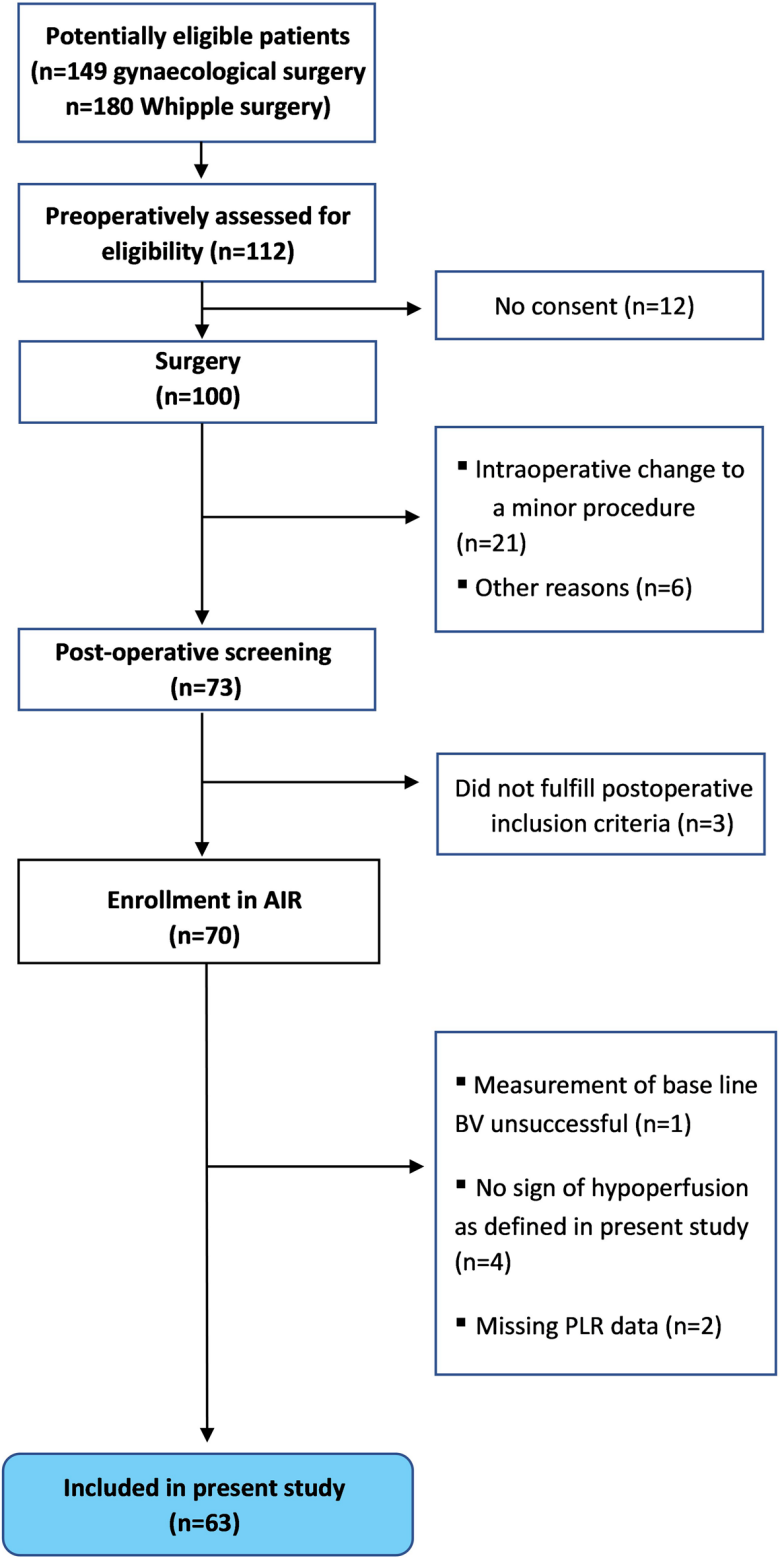
Flowchart of eligible, enrolled and analyzed patients

A total of 63 patients of the 70 patients included in the AIR trial were included in the present analysis (see Fig. 1 for flowchart of patients). Baseline characteristics and characteristics of the perioperative management can be seen in Table 1 and Appendix Table 3. The most common hypoperfusion inclusion criterion was lactate > 2 mmol/l (67%) followed by diuresis < 0.5 ml/kg/h (45%), hypotension (32%) and low ScvO_2_ (30%). Most patients expressed more than one sign of hypoperfusion (61%). Hemodynamic data and laboratory data prior to measurement of blood volume are presented in Table 2 and Appendix Table 4.

**Table 1:** Baseline characteristics and perioperative management

No. of patients	63
Age, years	68 (58–74)
Sex	60 (38)
Female, % (no.)	60 (38)
Male, % (no.)	40 (25)
Weight, kg	73 (62–86)
Height, cm	169 (164–176)
POSSUM physiology score	15 (13–17)
ASA	2 (2–3)
Type of surgery, % (no.)	
Whipple	54 (34)
Gynecological	46 (29)
Perioperative bleeding, ml	600 (300–1000)
Perioperative diuresis, ml/kg/h	0.7 (0.4–1.1)
Perioperative crystalloids, ml	4400 (4000–5250)
Perioperative colloid, ml	600 (425–1000)
Length of surgery, min	404 (329–497)
Length of anesthesia, min	503 (448–585)
Epidural anesthesia, % (no.)	92 (58)
Perioperative use of vasopressor, % (no.)	84 (53/63)
Perioperative use of inotropy, % (no.)	14 (9/63)

ASA, American Society of Anesthesiologists physical status; POSSUM, Physiological and Operative Severity Score for the enUmeration of Mortality and morbidity

Data presented as median (IQR) or percentage (no.)

**Table 2:** Hemodynamic data and laboratory data prior to plasma volume measurement

Heart rate, bpm	88 (74–95)
SBP, mmHg	114 (95–127)
MAP, mmHg	77 (66–87)
DAP, mmHg	57 (52–72)
CVP, mmHg	3 (0–8)
Lactate, mmol/l	1.4 (1.8–3.1)
Diuresis, ml/kg/h	0.6 (0.3–1.23)
Hematocrit	0.35 (0.32–0.38)
Hemoglobin, g/l	115 (103–124)

SBP, systolic blood pressure; DAP, diastolic arterial pressure; MAP, mean arterial pressure; CVP, central venous pressure

Parameters were measured in immediate proximity to plasma volume measurement

Data presented as median (IQR) except hematocrit which is described as fraction

### Blood volume and response to passive leg raising

**Fig. 2:**
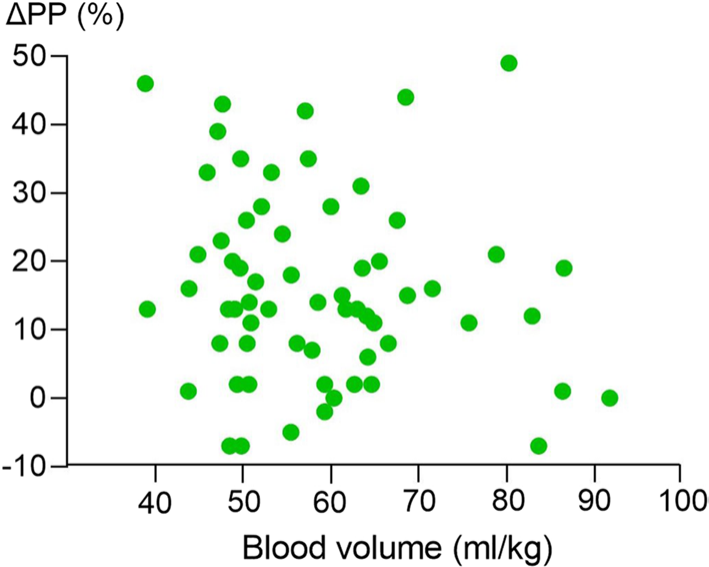
Change in pulse pressure (ΔPP) following a passive leg raising test in relation to blood volume

Median (IQR) plasma volume in the whole cohort was 2830 (2460–3495) ml. Blood volume was 4165 (3575–5153) ml, corresponding to 57 (50–65) ml/kg. Median change in pulse pressure after PLR was 14 (7–24) %; see Fig. 2. No correlation between blood volume and change in pulse pressure after PLR could be demonstrated (*r* = − 0.1459, 95% CI (− 0.3863 to 0.1131), *P* = 0.254). When analyzing each hypoperfusion criterion (elevated lactate, low blood pressure, low diuresis and low ScvO2) separately, no correlation between blood volume and change in pulse pressure was found in any of these subgroups (see Appendix Figs. 4, 5, 6, 7).

**Fig. 3:**
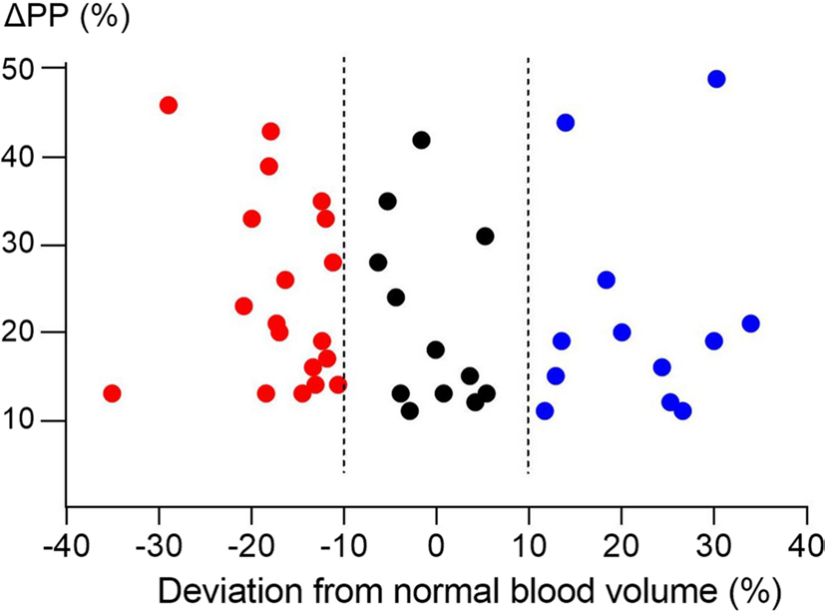
Change in pulse pressure (ΔPP) following a passive leg raising test and deviation of blood volume from normal blood volume. Dotted lines illustrate limits of euvolemia [13]

The change in pulse pressure was ≥ 9% in 68% (*n* = 43) of the patients and these patients were therefore deemed likely to be preload responsive. Of these patients, 44% (19/43) were hypovolemic, 28% (12/43) were euvolemic and 28% (12/43) were hypervolemic; see Fig. 3. In patients unlikely to be preload responsive (*n* = 20), 25% (5/20) were hypovolemic, 55% (11/20) were euvolemic and 20% (4/20) were hypervolemic. In a sensitivity analysis, we used a 15% deviation or more from predicted normal value to define hyper- and hypovolemia. Using these criteria in patients assumed likely to be preload responsive, 23% (10/43) were hypovolemic, 58% (25/43) were euvolemic and 19% (8/43) were hypervolemic. Among patients unlikely to be preload responders, 5% (1/20) were hypovolemic, 80% (16/20) were euvolemic and 15% (3/20) were hypervolemic.

## Discussion

This study on postoperative patients with clinical signs suggesting inadequate organ perfusion shows that about two thirds are likely to be preload responsive as judged by the response to passive leg raising. Less than half of the patients that were likely to be preload responsive were hypovolemic and a fourth were hypervolemic. No correlation between blood volume and change in pulse pressure was found.

To identify patients with inadequate organ perfusion, markers such as hypotension, low urine output, high lactate and low central venous oxygen saturation were used. Because these symptoms may be explained by mechanisms other than low cardiac preload, it could be argued that other methods or symptoms may have offered better discrimination in identifying patients with inadequate cardiac output. However, observational trials of fluid resuscitation practices have shown that the symptoms used in this study are common indications of fluid administration and hence identify a population in which fluid administration is likely to be considered in clinical reality [14].

To identify likely preload responders, we used a PLR test and change in pulse pressure. Patients had an arterial line according to unit protocol and therefore, pulse pressure was used as a pragmatic surrogate measure of change in cardiac output. We chose the 9% cut-off derived in one of the few studies including only spontaneously breathing patients [8]. In that study, this cut-off resulted in a sensitivity of 79% and a specificity of 85%. It should be noted that meta-analyses suggest that the use of change in pulse pressure to identify preload responders after passive leg raising has a sensitivity of only about 60% and specificity of 85% whereas measurement of cardiac output following passive leg raising has been shown to have a sensitivity of 83–88% and specificity of 80–92%. Taken together, this indicates that an important limitation of the present study is that the number of true preload responders may have been underestimated and that measurement of change in cardiac output following passive leg raising may have allowed for a better discrimination between responders and non-responders [5–7].

Accurate measurement of blood volume is dependent on blood volume remaining constant during the 10-min period from injection of tracer to blood sampling. This means that loss of ^125^I-HSA to the extravascular compartment due to ongoing bleeding or an increased microvascular permeability could have caused an overestimation of blood volumes [15]. Based on a median blood volume of 4.2 l in the whole cohort we estimate that a hypothetical bleeding of 500 ml/h will cause an overestimation in blood volume of about 2%. Because all patients remained stable for the 4-h observation period after the plasma volume measurement, we believe that blood loss of this magnitude is unlikely to have occurred. Moreover, we have previously reported that transcapillary escape rate of albumin (TER) in this cohort was normal [4]. Taken together we believe that these potential sources of error are unlikely explanations for the high incidence of hypervolemia.

How do we explain our finding that some hypervolemic patients are likely to be responders whereas some hypovolemic patients are unlikely to respond to fluid administration?

To find the answer, we must discuss the determinants of preload responsiveness—preload, cardiac contractility and afterload. *Preload* will dictate *where* on the Frank–Starling curve the heart is operating at, whereas *cardiac contractility* and *afterload* will affect the *slope* [2, 16, 17]. As mentioned in the introduction, preload is determined by the mean systemic filling pressure (P_msf_) and the right atrial pressure, the former being dependent on the stressed volume [2, 16, 17]. Based on this physiological framework, we can hypothesize that a combination of hypervolemia and preload responsiveness could be explained by several mechanisms. Firstly, venous vasodilation may decrease the stressed volume and result in a lower-than-normal P_msf_ and lower preload. This would increase the likelihood of preload responsiveness at a higher-than-normal blood volume. Such venous vasodilation could be secondary to epidural analgesia or surgery-induced inflammation [18, 19]. Secondly, arterial vasodilation may produce a lower-than-normal afterload, causing the heart to operate at the steep part of the curve, even at higher-than-normal preload. This could again be a consequence of the epidural analgesia and/or surgery-induced inflammation [18, 19]. In our study, peripheral vascular resistance was not measured, but if diastolic arterial pressure is used as a surrogate for arterial vascular tone, we can conclude that our participants most likely do not suffer from severe arterial vasodilation (< 50 mmHg) [20]. Conversely, the combination of hypovolemia and preload unresponsiveness may result from decreased cardiac contractility or increased afterload, leading to a flattened Frank–Starling curve which is unresponsive also at lower preload than normal. A potential decrease in cardiac contractility in our cohort may result from undiagnosed preoperative heart failure, negative inotropic effects of anesthetics or perioperative myocardial injury [21, 22]. Lastly the combination of hypovolemia and preload unresponsiveness could be a false negative response to the passive leg raise test. In a situation with pronounced venous vasodilation, it is possible that the volume of blood transferred from the peripheral to the central circulation during the passive leg raising test is insufficient to appreciably increase stressed volume and hence *P*_msf_ and preload.

In postoperative patients with signs of hypoperfusion, therapy aims at increasing tissue perfusion by increasing cardiac output. In a preload-responsive patient, this is commonly achieved by increasing preload by intravenous fluid administration. Assuming that our definition of hypervolemia is accurate, our results indicate that an increase in cardiac output by other means than fluid administration could be more physiological in a large fraction of preload-responsive postoperative patients. As suggested previously, such approaches may also have the potential to improve outcome by avoiding unnecessary fluid therapy [23–25].

One approach to increase preload without administration of fluid is to increase the stressed volume by administration of a venoconstrictor. As mentioned in the introduction and above, preload at a given right atrial pressure is determined by mean systemic filling pressure (*P*_msf_), which is dependent on the stressed venous volume. Alpha_1_-receptors are abundant in venous vessels and administration of an α_1_-receptor agonist such as noradrenaline may increase venous return by increasing the stressed venous volume and by that *P*_msf_^,^ [25–27]. The stressed and unstressed venous volumes cannot be measured in clinical practice, but support for such an effect of α_1_-receptor stimulation can be inferred from studies in which noradrenaline was suggested to dose dependently increase the filling pressure of the heart [25, 28]. Moreover, studies showing that the effect of PLR is decreased at increasing doses of noradrenaline align with the hypothesis that α_1_-receptor stimulation increases preload by an increase in the stressed venous volume [23, 29]. Depending on the clinical context, other approaches to increase cardiac output, such as administration of inotropic and/or vasodilatory drugs in order to increase contractility and/or decrease afterload, may be more appropriate.

At present, measurement of blood volume using our methodology is technically challenging, costly and time consuming. Based on this, we believe that novel methods for bedside determination of blood volume and/or stressed venous volume are required before the clinical benefit of interventions other than fluid administration to hypervolemic fluid responsive patients can be assessed.

### Strengths

The data for this post hoc analysis were derived from a randomized trial monitored by external monitors and plasma volume was measured using an established method by an investigator blinded to the response to the passive leg raise.

### Limitations

As mentioned above, the fact that we assessed fluid responsiveness by measuring change of pulse pressure after passive leg raise test is a limitation and direct measurement of change in cardiac output after a PLR, using for example transpulmonary thermodilution, may have resulted in better discrimination between preload responders and non-responders.

Although all efforts were made to minimize bias when designing the AIR trial and the present study, we acknowledge that this is a post hoc analysis of data with an inherent risk of bias. No power analysis was performed, and we cannot exclude that an association between blood volume and response to passive leg raising had been detected if sample size would have been larger. It should also be mentioned that blood volume can be regarded as a controlled parameter. That is, the clinician adjusts treatment to maintain what is perceived to be euvolemia and this probably limits the range of blood volumes observed in clinical practice. Moreover, it is possible that the fraction of preload-responsive patients with hypervolemia is dependent on intra- and post-operative care of the included patients and the results may not be applicable to patients with circulatory impairment in other settings, such as in the intensive care unit.

## Conclusions

A large fraction of postoperative patients with signs of hypoperfusion that are likely to be preload responsive are hypervolemic. In these patients, other therapeutic approaches than intravenous fluid administration may be a more rational approach to increase cardiac output.

## Figures and Tables

**Fig. 4:**
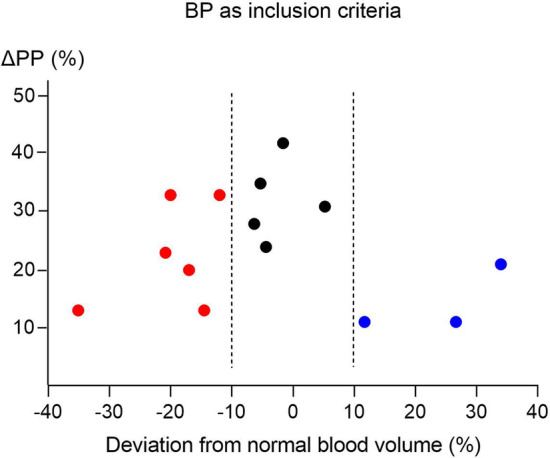
Change in pulse pressure (ΔPP) following a passive leg raising test in relation to blood volume, in patients included due to systolic BP < 100 or MAP < 65 mmHg. No significant correlation could be found (*r* = − 0.082 (95% CI − 0.598–0.483) *p* = 0.78, Spearman correlation coefficient). Dotted lines illustrate limits of euvolemia [13]

**Fig. 5:**
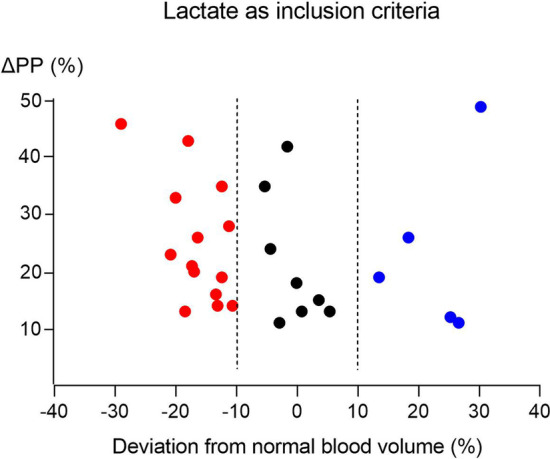
Change in pulse pressure (ΔPP) following a passive leg raising test in relation to blood volume, in patients included due to lactate level > 2 mmol/l. No significant correlation could be found (*r* = 0.312 (95% CI 0.627–0.0871), *p* = 0.11, Spearman correlation coefficient). Dotted lines illustrate limits of euvolemia [13]

**Fig. 6:**
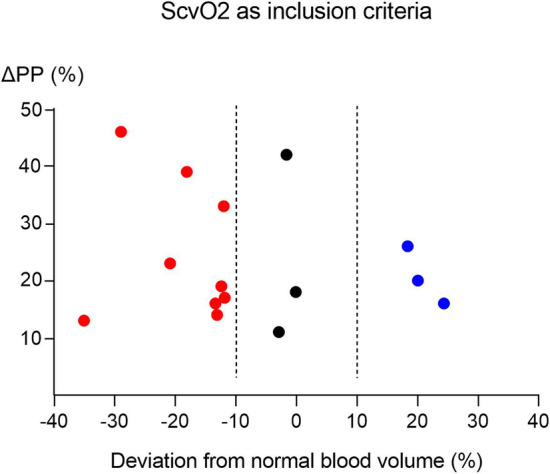
Change in pulse pressure (ΔPP) following a passive leg raising test in relation to blood volume, in patients included due to ScvO2 < 70%. No significant correlation could be found (*r* = − 0.054 (95% CI − 0.562–0.485), *p* = 0.85, Spearman correlation coefficient). Dotted lines illustrate limits of euvolemia [13]

**Fig. 7:**
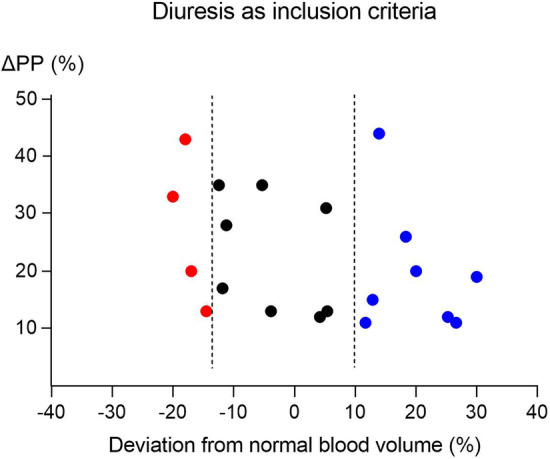
Change in pulse pressure (ΔPP) following a passive leg raising test in relation to blood volume, in patients included due to diuresis < 0.05 ml/kg/h. No significant correlation could be found. *r* = − 0.054 (95% CI − 0.562–0.485), *p* = 0.85, Spearman correlation coefficient). Dotted lines illustrate limits of euvolemia [13]

## Data Availability

The data are available from the corresponding author on reasonable request.

## Appendix

See Figs. 4, 5, 6, 7 and Tables 3, 4.

**Table 3:** ASA—American Society of Anesthesiologists physical status, POSSUM—Physiological and Operative Severity Score for the enUmeration of Mortality and morbidity

	All	Preload responders	Preload non-responders	Difference	*p*
Age, yrs	68 (58–74)	67 (59–73)	69 (59–74)	0 (− 5 to 4)	0.96
Female gender, % (no.)	60 (38/63)	46 (24/43)	70 (14/20)	N/A	0.28
Weight, kg	73 (62–86)	73 (63–86)	74 (61–85)	− 1.5 (− 9.0 to 8.0)	0.75
Height, cm	169 (164–176)	170 (165–177)	165 (162–174)	− 3.2 (− 8.0 to 1.7)	0.19
POSSUM physiology score	15 (13–17)	15 (9)	15 (11)	N/A	0.68
ASA	2 (2–3)	2 (2)	2 (1)	N/A	0.11
Perioperative bleeding, ml	600 (300–1000)	600 (300–1000)	500 (225–975)	− 50 (− 200 to 300)	0.61
Perioperative diuresis, ml/kg/h	0.7 (0.4–1.1)	0.7 (0.4–1.1)	0.4 (0.3–1.0)	0.2 (− 0.4 to 0.0)	0.12
Perioperative crystalloids, ml	4400 (4000–5250)	4350 (4000–5250)	4400 (3960–5440)	− 105 (− 861 to 650)	0.90
Perioperative colloid, ml	600 (425–1000)	750 (250–1000)	500 (500–1000)	− 56 (− 326 to 212)	0.68
Length of surgery, min	404 (329–497)	600 (300–1000)	399 (281–478)	− 32 (− 94 to 29)	0.29
Length of anesthesia, min	503 (448–585)	513 (454–592)	499 (360–562)	− 27 (− 90 to 33)	0.36
Epidural anesthesia, % (no.)	92 (58/63)	91(39/43)	95(19/20)	N/A	0.56
Perioperative use of vasopressor, % (no.)	84 (53/63)	86 (37/43)	80 (16/20)	N/A	0.54
Perioperative use of inotropy, % (no.)	14 (9/63)	14 (6/43)	15 (3/20)	N/A	0.91

Data are presented as median (IQR), percentage (no.), mean difference (95% CI) or median difference (95% CI) according to the Hodges-Lehmann method. Comparisons between preload responders and preload non-responders were performed using the Mann–Whitney, Student’s *t*-test or Fisher’s exact test as appropriate. No adjustments for multiple comparisons were made

**Table 4:** Parameters were measured in within 5 min of start of plasma volume measurement

	All	Preload responder	Preload non-responder	Difference	*p*
Heart rate, bpm	88 (74–95)	89 (71–95)	84 (76–94)	− 2 (− 11 to 8)	0.76
SBP, mmHg	114 (95–127)	115 (98–129)	106 (92–120)	6 (− 19 to 6)	0.29
MAP, mmHg	77 (66–87)	78 (68–92)	72 (65–80)	− 6 (− 14 to 3)	0.20
CVP, mmHg	3 (0–8)	2 (0–8)	4 (2–8)	1 (− 2 to 3)	0.66
Lactate, mmol/l	2.4 (1.8–3.1)	2.6 (1.9–3.1)	2.2 (1.7–2.9)	0 (− 1 to 0)	0.50
ScvO2, %	73 (68–76)	73 (68–77)	73 (67–75)	0.2 (− 0.9 to 0.4)	0.91
Hemoglobin, g/l	115 (103–124)	115 (102–123)	115 (111–126)	4 (− 4 to 12)	0.27
Hematocrit	0.35 (0.32–0.38)	0.35 (0.31–0.38)	0.36 (0.34–0.39)	− 0.01 (− 0.01 to 0.04)	0.28

Hematocrit described as fraction. Data are presented as median (IQR) or mean difference (95% CI)

Comparisons between preload responders and preload non-responders were performed using Student’s *t*-test. No adjustments for multiple comparisons were made

SBP, systolic blood pressure; MAP, mean arterial pressure; CVP, central venous pressure

## Abbreviations

BV: Blood volume
CO: Cardiac output
Hct: Hematocrit
HSA: Human serum albumin
IQR: Interquartile range
MAP: Mean arterial pressure
PLR: Passive leg raising
PV: Plasma volume
ScvO_2_: Central venous oxygen saturation
SD: Standard deviation
TER: Transcapillary escape rate
ΔPP: Change in pulse pressure
